# History of falls in patients with atrial fibrillation and risk of major outcomes: analysis from the Prospective GLORIA-AF Registry

**DOI:** 10.1007/s11357-025-01852-x

**Published:** 2025-08-28

**Authors:** Giulio Francesco Romiti, Bernadette Corica, Tommaso Bucci, Giuseppe Boriani, Brian Olshansky, Tze-Fan Chao, Menno V. Huisman, Marco Proietti, Gregory Y. H. Lip

**Affiliations:** 1 Liverpool Centre for Cardiovascular Science at University of Liverpool, Liverpool John Moores University and Liverpool Heart & Chest Hospital, Liverpool, UK; 2 Department of Translational and Precision Medicine, Sapienza – University of Rome, Rome, Italy; 3 Cardiology Division, Department of Biomedical, Metabolic and Neural Sciences, University of Modena and Reggio Emilia, Policlinico Di Modena, Modena, Italy; 4 Division of Cardiology, Department of Medicine, University of Iowa, Iowa City, USA; 5 Division of Cardiology, Department of Medicine, Taipei Veterans General Hospital, Taipei, Taiwan; 6 Institute of Clinical Medicine, and Cardiovascular Research Center, National Yang Ming Chiao Tung University, Taipei, Taiwan; 7 Department of Thrombosis and Hemostasis, Leiden University Medical Center, Leiden, The Netherlands; 8 Department of Clinical Sciences and Community Health, University of Milan, Milan, Italy; 9 Division of Cardiogeriatric Subacute Care, IRCCS Istituti Clinici Scientifici Maugeri, Milan, Italy; 10 Department of Clinical Medicine, Aalborg University, Aalborg, Denmark; 11 Medical University of Bialystok, Bialystok, Poland

**Keywords:** Atrial fibrillation, Falls, Oral anticoagulant, Outcomes

## Abstract

Falls pose concerns for the management and prognosis of patients with atrial fibrillation (AF). Epidemiological data on patients with AF who fall are limited. From the prospective global GLORIA-AF Registry Phase III study, we evaluated patients with a recent diagnosis of AF and CHA_2_DS_2_-VASc score ≥ 1 according to previous history of falling at baseline. We analyzed the associations with use of oral anticoagulant (OAC) and other drugs, risk of OAC discontinuation, and risk of major outcomes using multivariable regression models. Primary outcome was a composite of all-cause death and major adverse cardiovascular events (MACE); exploratory secondary outcomes were also analyzed. 20,875 patients (age 70.1 ± 10.3 years, 45.0% females) were included: 874 (4.2%) had prior falls. Patients with falls had more complex clinical profiles, were older, and were more likely female. No difference in OAC use was observed at baseline based on prior falls (OR [95%CI] 0.90 [0.75–1.08]), but patients with previous falls who received OAC were more likely treated with a NOAC compared to VKA (OR [95%CI] 1.29: 1.07–1.55) and were more likely to discontinue OAC during follow-up (HR 1.18, 95%CI 1.02–1.36). Patients with prior falls had a higher risk of the primary composite outcome (HR 1.63, 95%CI 1.40–1.90) and all secondary outcomes explored, including thromboembolism and major bleeding. Patients in the GLORIA-AF Registry with prior falls were more likely to receive a NOAC (vs. VKA) and had a higher risk of major adverse events, including all-cause mortality, thromboembolism, and major bleeding.

**Supplementary Information:**

The online version contains supplementary material available at 10.1007/s11357-025-01852-x.

## Introduction

As the incidence and prevalence of atrial fibrillation (AF) are increasing worldwide [1], patients with AF are increasingly older and more burdened by other cardiovascular and non-cardiovascular comorbidities [2]. A significant proportion of these patients present with physical disability [3], impaired mobility [4], and ultimately frailty [5, 6], which is in turn associated with worse outcomes [7–9]. Falls are common in the elderly and particularly in patients with impaired mobility and frailty, with 14 million older adults reporting falls in 2020 in the USA [10], resulting in increased morbidity, mortality, and healthcare associated costs [10, 11], as well as a detrimental impact on quality-of-life and daily activities, in part due to the fear of subsequent falls [12].

AF is associated with a higher risk of falls [13]. Patients with AF who fall have a risk of dire consequences (including bleeding) and present challenges in oral anticoagulant (OAC) management [14, 15]. Indeed, falls are associated with increased risk of long-term adverse events in patients with AF, including all-cause mortality, major bleeding, and intracranial hemorrhage [16, 17]. The perceived risk of falls (and fall-associated bleeding) represents a barrier to the use of OAC among physicians treating patients with AF [18]. Nevertheless, real-world epidemiological data on the factors associated with falls in patients with AF are limited; therefore, data on the impact of previous falling history on AF management and major outcomes are needed.

As such, from the prospective multinational *Global Registry on Long-Term Antithrombotic Treatment in Patients with Atrial Fibrillation* (GLORIA-AF) Registry Phase III, we analyzed associations of previous falls with management and clinical outcomes in patients with AF.

## Methods

We used data from the GLORIA-AF Registry, an international, prospective, multicenter registry program structured in 3 phases, aimed to evaluate real-world long-term efficacy and safety of dabigatran etexilate in AF patients. Complete details on the design, study procedures, and primary results of GLORIA-AF Registry are reported elsewhere [19–22]. For this analysis, we considered patients recruited during the phase III of the registry, performed between 2014 and 2016, in which adult patients (≥ 18 years) with a recent diagnosis of non-valvular AF (i.e., within 3 months or 4.5 months in Latin America) and a CHA_2_DS_2_-VASc score ≥ 1 were enrolled; patients included in this analysis were those with complete information on prior history of falls and the incidence of the primary composite outcome. Patients with AF due to a reversible cause, mechanical heart valve (or those expected to undergo valve replacement), previous treatment with VKA for > 60 days during their lifetime, other clinical indication for OAC, or short life expectancy (< 1 year) were excluded. Local institutional review boards approved the study protocol at each participating center, and all patients provided written informed consent. The study was conducted according to the Declaration of Helsinki and the Good Clinical Practice.

### History of falls, other comorbidities, and treatments

At baseline, data regarding demographics, comorbidities, and treatments received at baseline were collected by investigators for each patient enrolled by means of standardized electronic case report forms. Among the conditions recorded at baseline, study investigators were able to record if the patient had history of falling. No additional information on the timing and number of falls, as well as the severity of the episode(s), were routinely collected. Among treatments, for the purpose of this analysis, we considered antithrombotic use (i.e., use of OAC and type of OAC, either a vitamin K antagonist (VKA) or a non-vitamin K antagonist oral anticoagulant (NOAC)), dose of NOAC (either standard, reduced or other doses, as reported elsewhere [20]), interventional procedures (AF ablation and cardioversion), and other drugs received at baseline (i.e., angiotensin converting enzyme (ACE) inhibitors, angiotensin receptor blockers (ARB), diuretics, beta-blockers (either selective or non-selective), digoxin, verapamil/diltiazem, propafenone, flecainide, amiodarone, dronedarone, and other antiarrhythmic drugs).

### Follow-up and outcomes

In phase III of the GLORIA-AF Registry, patients underwent a 3-year follow-up. For this analysis, we evaluated:

- All-cause mortality
- Major adverse cardiovascular events (MACE; defined as the composite of cardiovascular death, stroke, and myocardial infarction)
- Thromboembolism (defined as a composite of stroke, transient ischemic attack (TIA), and non-central nervous system thromboembolism)
- Major bleeding (defined as a life-threatening or fatal bleeding, symptomatic bleeding in a critical organ, or a bleeding associated with a hemoglobin reduction of ≥ 20 g/L or leading to ≥ 2 units of blood transfusion)

Our *primary outcome* for this analysis was the composite of all-cause death and MACE. The other outcomes were considered as exploratory secondary outcomes.

### Statistical analysis

Continuous variables were reported as either mean ± standard deviation (SD) or median and interquartile range (IQR) and compared with parametric or non-parametric tests, respectively. Categorical variables were reported as frequencies (percentages) and compared with chi-square test.

We assessed characteristics associated with history of falling at baseline using a multiple logistic regression model, which included components of the of CHA_2_DS_2_-VASc score (congestive heart failure, arterial hypertension, age (< 65, 65–75, or ≥ 75 years), diabetes, history of stroke/TIA, coronary artery disease (CAD), peripheral artery disease (PAD), and sex), region of recruitment, type of AF (paroxysmal, persistent, or permanent), history of previous bleeding and dementia, and body mass index (BMI), systolic blood pressure (SBP), and diastolic blood pressure (DBP) at baseline.

Linear association of continuous variables with history of falls was assessed at the univariable level. If non-linearity was observed, variables were included in the multivariable model as restricted cubic splines with 4 knots at default placement; otherwise, they were modeled as linear. Results of the multivariable models were reported as odds ratio (OR) and 95% confidence intervals (CI).

Odds of receiving treatment at baseline according to the history of falls were analyzed using multiple logistic regression models, adjusted for components of the CHA_2_DS_2_-VASc, type of AF, history of bleeding, and BMI. Results were reported as OR and 95%CI. Association of history of falling with risk of OAC discontinuation (defined as a switching to another antithrombotic regimen, including different OAC, or an interruption longer than 30 days of the treatment received at baseline [23]) at 2 year of follow-up was also explored through multiple-adjusted Cox regression model, adjusted for the same set of covariates; results were reported as hazard ratio (HR) and 95%CI.

The risk of major outcomes according to history of falls at baseline was analyzed using multiple-adjusted Cox regression models, adjusted for the components of the CHA_2_DS_2_-VASc score, type of AF, BMI, history of bleeding, and use of OAC. Results were reported as HR and 95%CI. For the primary outcome, Kaplan–Meier curves were also reported, and survival distributions were compared with log-rank test. Finally, for the primary outcome, we explored whether the association of prior falls with risk was modified across several baseline characteristics (i.e., age, sex, geographical location, type of AF, use of OAC, CHA_2_DS_2_-VASc score group, history of stroke/TIA, heart failure, coronary artery disease, and history of previous bleeding), by modeling two-way interactions.

A two-sided *p* < 0.05 was considered statistically significant. All analyses were performed using R 4.3.1 (R Core Team 2020, Vienna, Austria).

## Results

We included 20,875 patients (age 70.1 ± 10.3 years, 45.0% females) who were enrolled in the GLORIA-AF Registry Phase III. History of falls was reported in 874 (4.2%) of patients. Baseline characteristics according to the presence of history of falls at baseline are reported in Table 1. On average, patients with history of falling were older (75.2 ± 9.2 vs. 69.9 ± 10.3 years, *p* < 0.001), more likely female (56.5% vs. 44.5%, *p* < 0.001) and had a higher prevalence of several comorbidities, including arterial hypertension, and isolated systolic hypertension (defined as baseline systolic blood pressure ≥ 140 mmHg and baseline diastolic blood pressure < 70 mmHg), as well as coronary artery disease, diabetes mellitus, history of stroke/TIA, and history of bleeding. History of falls was more frequently reported in patients recruited in North America and less frequent in patients recruited in Asia; moreover, the prevalence of history of falling increased with age of patients (2.1% in patients < 65 years; 3.1% in patients 65–74 years; 6.6% in patients ≥ 75 years; *p* < 0.001) and was higher in patients with paroxysmal AF (4.5%) compared to those with persistent AF or permanent AF (3.7% and 4.0%, respectively; *p* = 0.014). Patients with prior falls had also higher CHA_2_DS_2_-VASc (3.9 ± 1.6 vs. 3.1 ± 1.5, *p* < 0.001) and HAS-BLED (1.7 ± 0.9 vs. 1.4 ± 0.9, *p* < 0.001) scores.

**Table 1:** Baseline characteristics according to history of falls at baseline

Variables	No history of falls (*n* = 20,001)	History of falls (*n* = 874)	*p*
Age, mean (SD)	69.9 (10.3)	75.2 (9.2)	< 0.001
Female sex, *n* (%)	8892/20,001 (44.5)	494/874 (56.5)	< 0.001
BMI, median [IQR]	27.5 [24.4, 31.4]	27.7 [24.4, 31.7]	0.435
Systolic blood pressure at baseline, mmHg, median [IQR]	130 [120–142]	132 [120–145]	0.022
Diastolic blood pressure at baseline, mmHg, median [IQR]	80 [70–85]	75 [68–83]	< 0.001
**Region, *n* (%)**			< 0.001
North America	4735/20,001 (23.7)	333/874 (38.1)	
Europe	9664/20,001 (48.3)	424/874 (48.5)	
Asia	4024/20,001 (20.1)	77/874 (8.8)	
Other	1578/20,001 (7.9)	40/874 (4.6)	
**AF type, *n* (%)**			0.014
Paroxysmal AF	11,225/20,001 (56.1)	533/874 (61.0)	
Persistent AF	6862/20,001 (34.3)	261/874 (29.9)	
Permanent AF	1914/20,001 (9.6)	80/874 (9.2)	
**Symptoms, *n* (%)**			0.026
EHRA III-IV	6165/20,001 (30.8)	301/874 (34.4)	
**Medical history, *n* (%)**			
Hypertension	14,876/19,978 (74.5)	695/872 (79.7)	0.001
Isolated systolic hypertension*	446/19,848 (2.2)	41/871 (4.7)	< 0.001
Heart failure	4350/19,875 (21.9)	176/868 (20.3)	0.279
CAD	3690/19,583 (18.8)	190/852 (22.3)	0.013
Diabetes mellitus	4633/20,001 (23.2)	230/874 (26.3)	0.034
PAD	560/19,870 (2.8)	46/868 (5.3)	< 0.001
Previous stroke/TIA	2800/20,000 (14.0)	184/874 (21.1)	< 0.001
Previous bleeding	989/19,829 (5.0)	123/860 (14.3)	< 0.001
Chronic obstructive pulmonary disease	1157/19,944 (5.8)	95/869 (10.9)	< 0.001
Abnormal kidney function^§^	358/19,808 (1.8)	21/864 (2.4)	0.227
Dementia	98/19,944 (0.5)	22/867 (2.5)	< 0.001
History of cancer	1929/19,847 (9.7)	150/863 (17.4)	< 0.001
**Risk scores**			
CHA_2_DS_2_-VASc, mean (SD)	3.1 (1.5)	3.9 (1.6)	< 0.001
HAS-BLED, mean (SD)	1.4 (0.9)	1.7 (0.9)	< 0.001

Legend: *BMI*, body mass index; *CAD*, coronary artery disease; *EHRA*, European Heart Rhythm Association; *IQR*, interquartile range; *PAD*, peripheral artery disease; *SD*, standard deviation; *TIA*, transient ischemic attack. *Isolated systolic hypertension defined as systolic blood pressure at baseline ≥ 140 mmHg and diastolic blood pressure at baseline < 70 mmHg. ^§^Abnormal kidney function defined as chronic dialysis, renal transplantation, or serum creatinine ≥ 2.26 mg/dl

### Factors associated with history of falls

**Fig. 1:**
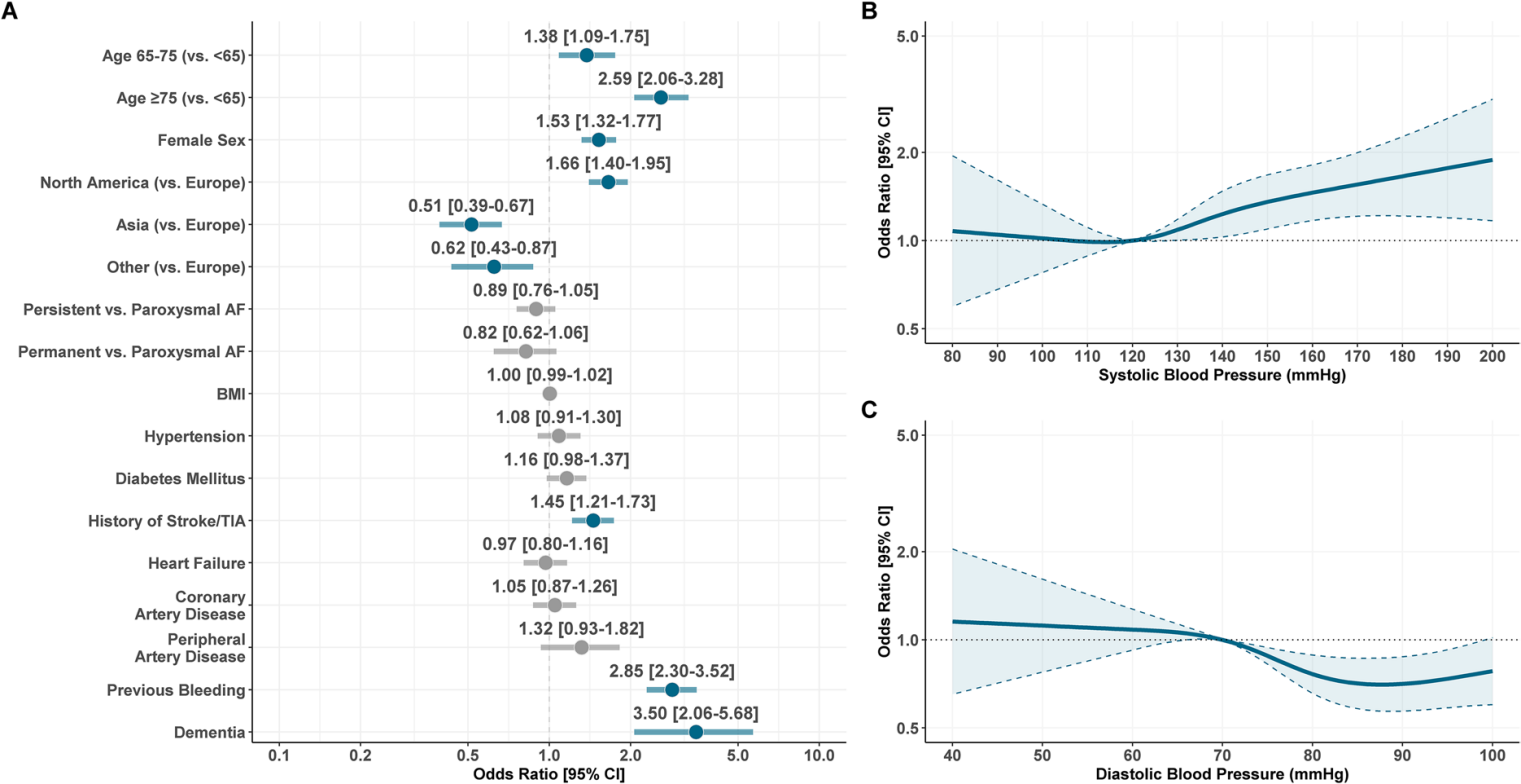
Factors associated with history of falls at baseline. Legend: **A** categorical variables and BMI; **B** systolic blood pressure; **C** diastolic blood pressure. AF, atrial fibrillation; BMI, body mass index; CI, confidence interval; TIA, transient ischemic attack

The results of the multiple-adjusted logistic regression model on factors associated with history of falls at baseline are reported in Fig. 1. Increasing age, female sex, and recruitment in North America were associated with an increased odds of presenting with history of falling, as well as history of stroke/TIA (OR 1.45, 95%CI 1.21–1.73), history of previous bleeding (OR 2.85, 95%CI 2.30–3.52), and dementia (OR 3.50, 95%CI 2.06–5.68). Among continuous variables, we observed an increase in the odds of previous falling with higher systolic blood pressure and lower odds for diastolic blood pressure > 70 mmHg (Fig. 1).

### Association with treatments

**Fig. 2:**
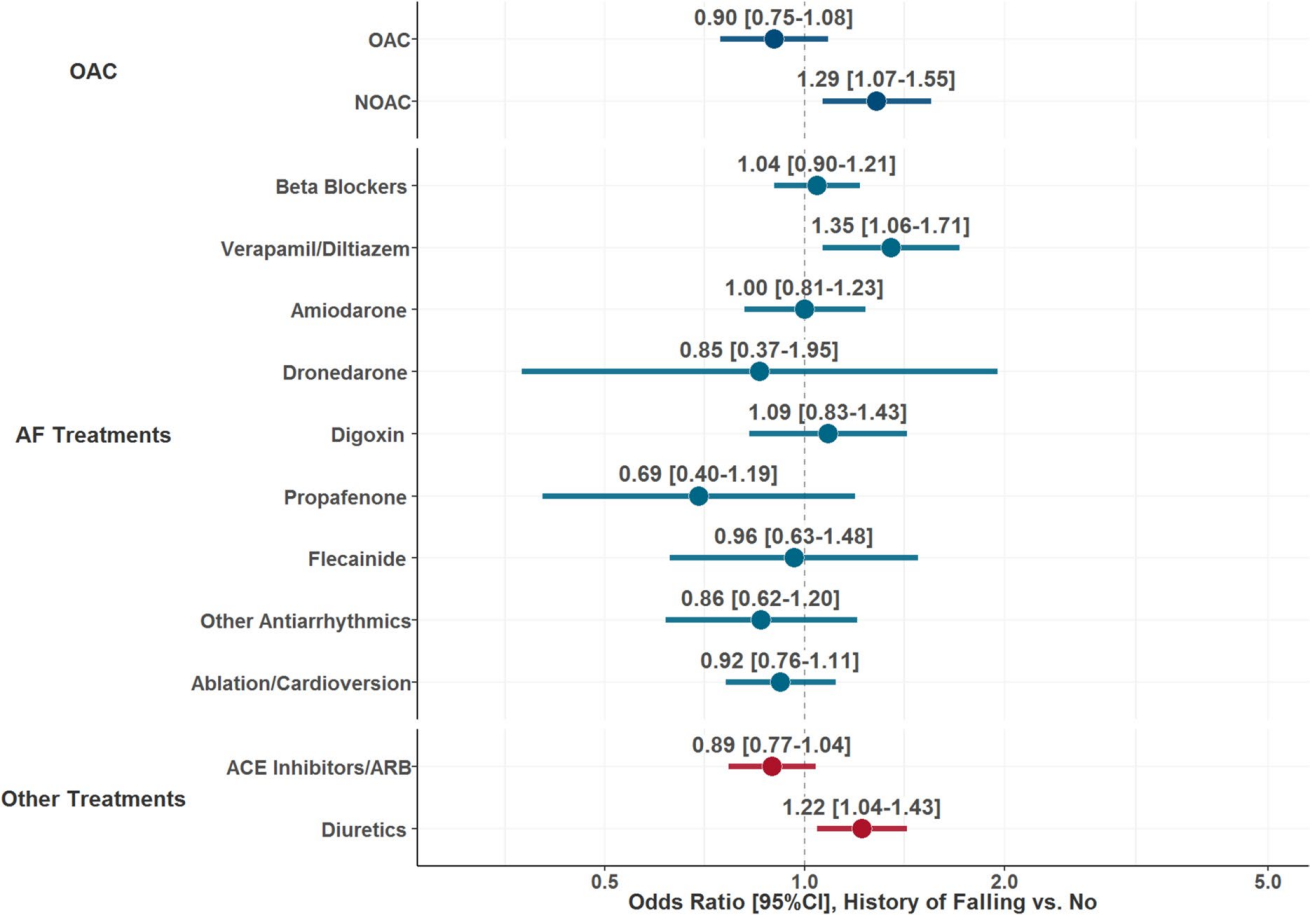
Association of history of falls with treatment at baseline. Legend: ACE, angiotensin converting enzyme; ARB, angiotensin receptor blockers; AF, atrial fibrillation; NOAC, non-vitamin K antagonist oral anticoagulant; OAC, oral anticoagulant

Treatments according to history of falls are reported in Table S1. Patients with and without history of falls had similar rates of OAC use at baseline (81.8% vs. 82.2%, respectively); however, patients with prior falls more frequently received NOACs (63.0% vs. 59.2%, and less frequently received a VKA (18.8% vs. 23.0%) compared to patients without prior falls. Moreover, among those who received a NOAC, reduced-dose NOACs were more commonly used in patients with previous falls compared to those without falls (34.5% vs. 29.1%, *p* = 0.020). On multivariable adjusted logistic regression analysis, no difference was observed for OAC vs. no OAC use according to history of falls at baseline (OR 0.90, 95%CI 0.75–1.08); conversely, patients who had prior falls presented higher odds of receiving NOAC over VKA (OR 1.29, 95%CI 1.07–1.55). Differences were observed also for other drugs (Fig. 2).

Among patients who received OAC at baseline, OAC discontinuation occurred more frequently at 2 years in patients with history of falls at baseline (29.7% vs. 27.2%; Fig. S1); at multivariable adjusted Cox-regression analysis, history of falls was marginally associated with a higher hazard of discontinuing OAC at 2 years (HR 1.18, 95%CI 1.02–1.36).

### Association with clinical outcomes

**Fig. 3:**
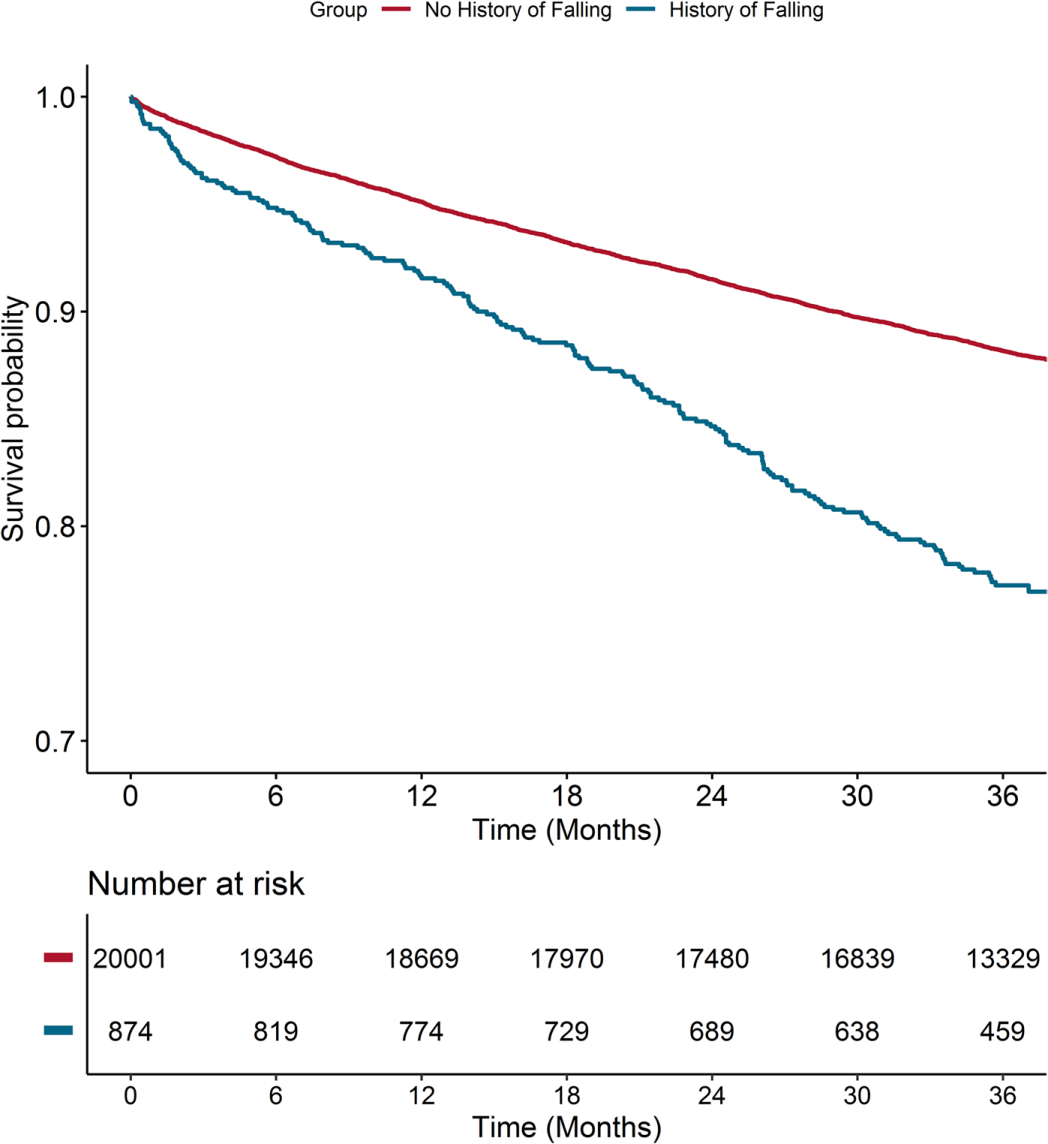
Kaplan–Meier curves for the primary composite outcome of all-cause death and MACE according to history of falling at baseline. Legend: log-rank 89.2, *p* < 0.001

During a median follow-up of 3.0 (IQR 2.9–3.1) years, the cumulative incidence of the primary composite outcome was higher in patients with history of falls (Fig. 3; *p* < 0.001). On multivariable adjusted Cox-regression analysis, patients with history of falls had a higher hazard of the primary composite outcome (HR 1.63, 95%CI 1.40–1.90, Table 2). Similar results were observed for exploratory secondary outcomes, with history of falls associated with a higher risk of all-cause death (HR 1.78, 95%CI 1.50–2.10), MACE (HR 1.43, 95%CI 1.14–1.80), thromboembolism (HR 1.58, 95%CI 1.20–2.10), and major bleeding (HR 1.81, 95%CI 1.38–2.39).

**Table 2:** Multiple Cox regressions on the risk of major outcomes according to history of falling at baseline

	**Incidence rate per 100 persons-years [95%CI]**	**Adjusted hazard ratio [95%CI]**^†^	***p***
**Primary outcome**			
*Composite of all-cause death and MACE*			
No history of falls	4.2 [4.1–4.4]	Ref	
History of falls	8.6 [7.4–9.9]	**1.63 [1.40–1.90]**	** < 0.001**
**Secondary outcomes**			
*All-cause death*			
No history of falls	3.2 [3.0–3.3]	Ref	
History of falls	7.0 [6.0–8.2]	**1.78 [1.50–2.10]**	** < 0.001**
*MACE*			
No history of falls	2.3 [2.1–2.4]	Ref	
History of falls	3.9 [3.2–4.9]	**1.43 [1.14–1.80]**	**0.002**
*Thromboembolism*			
No history of falls	1.3 [1.2–1.4]	Ref	
History of falls	2.7 [2.0–3.4]	**1.58 [1.20–2.10]**	**0.001**
*Major bleeding*			
No history of falls	1.2 [1.1–1.3]	Ref	
History of falls	2.8 [2.1–3.6]	**1.81 [1.38–2.39]**	** < 0.001**

Legend: ^†^Adjusted for age class, sex, type of AF, BMI, arterial hypertension, diabetes, heart failure, coronary artery disease, peripheral artery disease, previous stroke/TIA, previous bleeding, and use of OAC. Bold text depicts statistically significant results at *p* < 0.05 level. *CI*, confidence intervals; *IR*, incidence rate; *Ref.*, reference

**Fig. 4:**
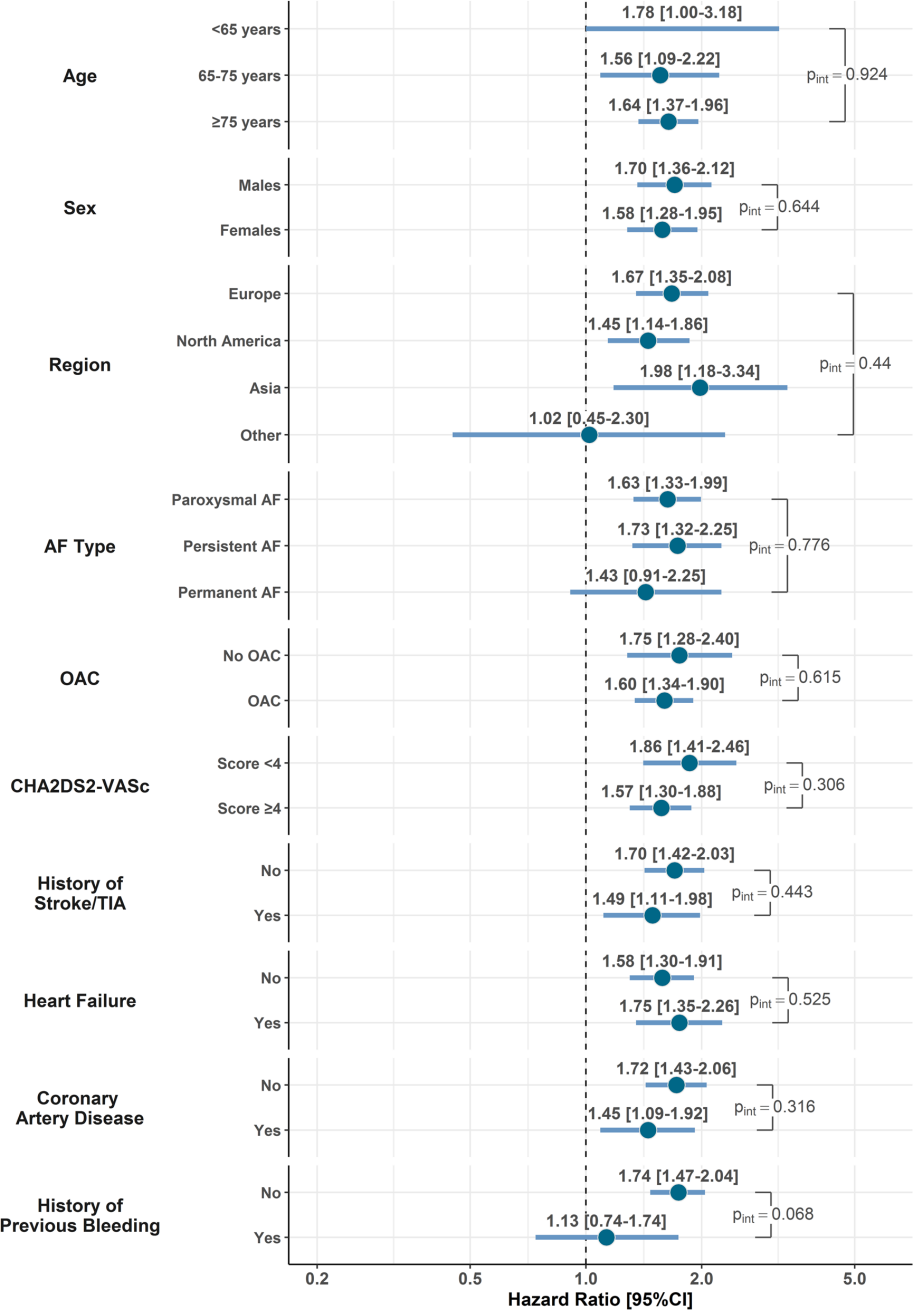
Interaction analysis on the risk of the primary outcome according to history of falling at baseline. Legend: AF, atrial fibrillation; OAC, oral anticoagulant; TIA, transient ischemic attack

When we analyzed the interaction of history of falling and key clinical characteristics on the risk of the primary outcome, we did not observe any statistically significant interaction across groups of age, sex, geographical region, type of AF, use of OAC at baseline, CHA_2_DS_2_-VASc score (≥ 4 vs. < 4), history of stroke/TIA, heart failure, and coronary artery disease. We observed some evidence for a higher magnitude of association of history of falls with primary outcome in patients without previous history of bleeding, although non-statistically significant (p_int_ = 0.068) (Fig. 4).

## Discussion

These data from a global, contemporary registry of patients with AF indicate: (1) history of falls is common in patients with AF, with prevalence that differs according to geographical region, and is associated with key clinical characteristics, including age, female sex, history of previous thromboembolic or bleeding events, and dementia; (2) patients with history of falls are more likely treated with NOAC over VKA and marginally more likely to discontinue OAC during follow-up; (3) history of falling is associated with worse prognosis during follow-up, even after adjusting for potential confounders, with an increased risk of all outcomes investigated; and (4) the association was not modified by key clinical characteristics, as shown by our exploratory interaction analysis.

Our results are in accordance with those from the ARISTOTLE trial, in which 4.6% of patients enrolled had a previous history of falling in the preceding year [17]. Other observational studies found broadly similar estimates, ranging from 1.1% to 7.4% [16, 24]. These figures should be interpreted with caution, as they may underpin potential under-reporting of previous falling in patients with AF. Indeed, previous studies suggested that falls may be under-reported by patients (particularly when asked at a distance from the event) [25, 26], and prospective studies report an incidence of falls in patients with AF up to 5%/year [27], and even higher in other cohorts [28, 29]. Differences in healthcare systems, including reimbursement policies, may also explain some of the geographical differences found (with history of falls more frequently reported in North American patients, and less likely in patients recruited in Asia); therefore, while our estimates are in line with previous observations, the prevalence of previous falls may be even higher in patients with AF. The AF-fall relationship, which is sustained by a number of direct and indirect mechanisms [13], is also strengthened by evidence of higher risk of falling in patients with vs. without AF, as shown by a previous meta-analysis [13]. This relationship is likely fostered by factors common to both AF and fall risk, including aging, frailty [7, 30], multimorbidity [27, 31], and cognitive impairment [32, 33].

Consistently, we found that older age and other key clinical characteristics (including previous stroke/TIA, previous bleeding, and dementia) were associated with prior falls. These results appear in accordance with the hypothesis that reduced mobility and impairment in physical function (which can be associated with aging and previous ischemic events) may increase the risk of falling. Nevertheless, falls may also be responsible for previous bleeding events [17, 24], thus explaining the association observed. Dementia and cognitive impairment have been associated with falling [34], with a bidirectional relationship being described [32]. Finally, our results on blood pressure levels, while needing cautious interpretation, may also reflect the role of uncontrolled hypertension and orthostatic hypotension in the risk of falls [35], although we were unable to analyze the contribution of these specific factors in this study.

While we did not observe differences in use of OAC at baseline, previous falls were associated with a higher odds of receiving NOACs compared to VKA on multiple regression analysis. The higher use of NOACs reflects the historical trends observed in the general AF population [36], but other factors may also explain the observed higher use of NOACs in patients with prior falls. Indeed, NOACs are associated with a lower risk of bleeding (and particularly intracerebral hemorrhage) than VKA [37], making them an appealing option for patients with prior falls, as well as those who have a high bleeding risk. Moreover, a history of prior bleeding was approximately three times more common among patients with prior falls in our study (14.3% vs. 5.0%). This higher risk of bleeding may partly explain the higher use of NOACs that we observed.

Our findings are consistent with previous studies showing better safety of NOACs over VKA in patients with history of falls [16], including a meta-analysis (in patients at risk for falling) [38], and a sub-analysis of the ARISTOTLE trial, that confirmed the greater safety of apixaban regarding major bleeding and intracranial bleeding, even in patients with previous falls [17]. We also observed a higher use of reduced-dose NOACs compared to standard-dose NOACs in patients with previous falls; this finding may be explained by a higher prevalence of NOAC dose reduction criteria, as well as by concerns in the bleeding risk of patients with prior falls.

Notwithstanding the similar use of OAC at baseline, we observed a marginally higher risk of OAC discontinuation—perhaps driven also by the higher risk of major bleeding observed during follow-up—that highlights the challenges associated with the long-term management of these patients. Although we were unable to analyze it in our study, the subsequent risk of falls (which was likely higher in patients with a previous history of falls) may have also influenced the discontinuation of OAC and, in turn, the risk of other events—including thromboembolic events. Moreover, patients with history of falls were less likely to have received ablation or cardioversion and more likely treated with verapamil/diltiazem (which are used for rate control).

Several hypotheses can explain these differences in AF management, including the perceived complexity of patients who fall, and thus a potential lower expected benefit from interventional rhythm control strategies, notwithstanding a numerically higher proportion of them presenting with higher symptoms at baseline. Overall, our results may also reflect the uncertainties on the potential benefit and outcomes in older and more complex patients undergoing interventional procedures, such as AF ablation [39, 40].

We also found that history of falls was associated with a higher risk of all outcomes investigated, including our primary outcome of all-cause death and MACE, thromboembolism, and major bleeding. The association with the primary outcome was also not different across key relevant subgroups of patients. While the detrimental effect of history of falls on all-cause mortality and bleeding was already observed, uncertain findings were reported on the association with thromboembolic events [16, 17, 41]; conversely, we report an increased risk of thromboembolism in patients with history of falls. Different baseline characteristics (and thromboembolic risk) of our patients, and longer follow-up times, may contribute to explain these results, which should be however interpreted with caution.

Taken together, our findings have important clinical implications. The higher risk of all major outcomes investigated in patients with history of falls remarks the complexity and clinical unmet needs of these patients. The association of previous falling with worse prognosis may be both direct (e.g., through increasing the risk of subsequent falls and therefore adverse events) and indirectly mediated by the overall complexity and clinical risk profile of these patients. As shown in our study, clinical characteristics entailing a more complex profile (e.g., age, history of previous stroke/TIA and bleeding, and also dementia) were associated with history of falls at baseline, and such factors have been previously used to identify patients at higher risk of worse outcomes during follow-up [42–45].

Our results suggest that a previous history of falling may represent an indicator of higher risk in real-world patients with AF, prompting more efforts to improve outcomes. These include specific interventions to reduce and mitigate risks of subsequent falls [46, 47] and rational decisions on OAC therapy and potential alternatives (such as left atrial appendage occlusion) in specific subgroup of patients with AF, considering also the increased risk of thromboembolic events (as we found in these patients) [48]. Such interventions and decisions should be made in the framework of an integrated and comprehensive approach to the treatment of AF, as recommended by international guidelines on AF [49–52]; in this context, the “Atrial fibrillation Better Care” (ABC) pathway has been found associated with better outcomes even in patients with complex clinical profiles [53–56] and can be used to effectively streamline such an holistic or integrated approach even in patients with complex health needs, as those with high risk of falls.

### Strength and limitations

Our study is based on a contemporary, global and large cohort of patients with AF, thus representing a comprehensive analysis on the association of history of falls with the natural history of real-world patients with AF. Notwithstanding this, we acknowledge some limitations. First, we did not have information regarding the date, number, and severity of the previous episodes of falls, and subsequent falls occurring during follow-up were not among the outcomes routinely collected in the study. Therefore, we were unable to consider and analyze these factors in our study. We cannot also exclude a potential underreporting of history of falls in our cohort, as previously discussed. Moreover, we were unable to consider other potential risk factors for falling (including orthostatic hypotension, osteoporosis, fractures, and immobilization) and to evaluate their impact on the risk of major outcomes, as well as the impact of some drugs (such as hypertensive treatment) on the risk of subsequent falls during follow-up. As this was a post hoc analysis of a prospective observational registry, we may have had limited power to detect differences in some comparisons for patients with vs. without history of falls, including in the interaction analyses on the risk of the primary outcome. Also, although we adjusted our regression analyses for several factors, we cannot exclude the contribution of other unaccounted confounders in the results observed. Therefore, our findings should be interpreted with caution. Finally, our results on secondary outcomes were not adjusted for multiple comparisons, and as such should be regarded as exploratory and interpreted with caution.

## Conclusions

In patients with AF, history of falling is associated with different management (including choices on type of OAC) and a higher risk of major adverse events, including all-cause mortality, thromboembolism, and major bleeding. Patients with history of falls have complex clinical profiles that require tailored and integrated management approaches.

## Supplementary Information

Below is the link to the electronic supplementary material.

Supplementary file1 (DOCX 355 KB)

## Data Availability

Data supporting this study by the data contributors Boehringer Ingelheim were made and are available through Vivli, Inc. Access was provided after a proposal was approved by an independent review committee identified for this purpose and after receipt of a signed data sharing agreement.
